# Tanner on Infancy and Childhood

**Published:** 1871-09

**Authors:** 


*Tanner on Infancy and Childhood.* By MEADOWS. Third American Edition. Philadelphia: Lindsay and Blakiston, 1871.

Revised and improved works upon the diseases of Infancy and Childhood, are, perhaps, of as great value to the profession and mankind as are any books upon Medicine and Surgery. It is obvious to all extensive observers, that the diseases of Infancy and Childhood, though for the most part simple and easily known, are yet imperfectly studied, and their importance but partially understood. It is remarkable that with the care young humanity receives, that so many arrive at adult life; and when we consider what hygienic conditions attend the poorer classes in large cities, we can only look with astonishment, making ourselves almost believe that the general laws of health are altogether suspended, or even changed so far as children are concerned—that they are healthier the poorer they are fed and clothed, and the dirtier they are kept. It appears to us that this book is written upon very good common sense principles, and that with all that is known of the causes pathology symptoms and treatment of the numerous diseases of infancy and childhood is gained that comprehensive understanding of the general laws which underlie this young life, as to make this book of value to all attentive readers. It is concise, comprehensive, and complete in its teachings, and will, we believe, continue to hold rank as one of the best standard works upon this subject.

We should be glad to speak more in detail of its merits, and to show by quotation its teachings, but space will not permit. Our readers must accept our assurance that it is a good work upon subjects too much neglected.

